# Working Right Ways in Foot Health With and for First Nations Peoples: Research Method Guided and Governed by First Nations Ways of Knowing, Being, and Doing in Cross‐Sectional Qualitative Study Design

**DOI:** 10.1002/jfa2.70131

**Published:** 2026-02-23

**Authors:** James Gerrard, Shirley Godwin (Badimaya Yamatji), Kim Whiteley (Wiradjuri), James Charles (Kaurna), Sean Sadler, Vivienne Chuter

**Affiliations:** ^1^ Discipline of Podiatry School of Health Sciences Western Sydney University Sydney New South Wales Australia; ^2^ Wardliparingga Aboriginal Health Equity Unit South Australian Health and Medical Research Institute Adelaide South Australia Australia; ^3^ La Trobe University Rural Health School Bendigo Victoria Australia; ^4^ Remote Area Health Corps Canberra New South Wales Australia; ^5^ First Peoples Health Unit Griffith University Brisbane Queensland Australia; ^6^ University of Newcastle Gosford New South Wales Australia

**Keywords:** co‐design, cultural safety, First Nations Peoples (Australia), foot health, participatory action research, systemic racism

## Abstract

**Background:**

Underpinning ongoing colonisation of the lands now known as Australia, scientific racism in colonial research delivered flawed results by building Indigenous inferiority into methodology to produce dehumanising conclusions of First Nations Peoples. Scientific racism facilitated exclusion of First Nations Peoples from systems design and development; foregrounding ways exclusive and enforced colonial health systems cause First Nations health and wellbeing inequality. Inequities in foot health contribute to this inequality. This work describes and documents a process of First Nations‐led authentic co‐design for foot health research. This study represents ways and means to develop culturally responsive foot health research as judged by First Nations Peoples which will translate into improved and more responsive ways of delivering foot care.

**Methods:**

Non‐Indigenous and First Nations Peoples sought authentic First Nations‐led co‐design process in foot health research methods, a governing First Nations Advisory Group, and broader First Nations governance and ethics approvals. Indigenous methodology, data sovereignty, and redistribution of power were imperative in ways of working. First Nations‐led co‐design developed culturally responsive semi‐structured interviews to collect data. Talking with 10 registered health practitioners who work closely with lower limb and foot health represented the right mix of participants and enough data to convey a more complicated mosaic of multi‐faceted stories. First Nations expertise informed analytic induction and the use of inductive reasoning and constant comparison to identify common and overarching themes, and to perform thematic analysis.

**Results:**

Authentic First Nations‐led ways of working in cross‐sectional qualitative study design are documented. Results of data analysis following these ways of working will be published subsequently.

**Conclusion:**

This work provides insights into working right ways in research which will underpin good foot health services with and for First Nations Peoples. The paper highlights ways of working that empowers First Nations Peoples in authentic co‐design. First Nations‐led foot health research changes ways of working to counter inequities in foot health caused and maintained by ongoing colonisation and systemic racism. This study provides qualified voiced lived experience which foot health researchers must listen to and receive learning and direction from.

**Abbreviations**
AH & MRC: Aboriginal Health and Medical Research Council
AIATSIS: Australian Institute of Aboriginal and Torres Strait Islander Studies
IREG: Indigenous Research Ethics Guidelines
NHMRC: National Health and Medical Research Council
PAR: Participatory Action Research

## 1 Note

Nomenclature: This work includes the nomenclature; Aboriginal and Torres Strait Islander Peoples, First Nations Peoples, and Indigenous Peoples. Neither singularly nor collectively do they adequately represent the immense diversity of language groups and cultural values across this continent's Traditional Custodians and Sovereign Owners [1, 2]. The authors privilege ways of using terminology that are self‐determined, that communicate diversity and sovereignty, and that minimise use of terms that are imposed. Non‐Indigenous people is the language used to represent and be inclusive of Australians who are not First Nations Peoples [3]. The terms decolonise, decolonisation, and decolonising methodology throughout this work describe being inclusive of First Nations worldviews and holistic conceptualisations of health and well‐being [4], whilst challenging and de‐centring dominant colonial views and divesting colonial power [5]. The term method is very much a Western academic term and is described in this publication as ways of working, not just inclusive of, but led by First Nations ways of knowing, being, and doing.

History: The authorship acknowledges that since invasion, Australia's healthcare system has been dismissive of an ongoing and successful First Nations health paradigm in place since time immemorial.

Statistics: When dealing with statistics, pathology, and reported levels of health, authors acknowledge the important considerations and groundings within which to position discussions that respects the strength and pride of First Nations Peoples [6]. The research does not include deficit discourse, does not assign First Nations Peoples as a vulnerable population, and does not present statistics to obscure the root causes of health inequities [6], namely colonisation and systemic racism [6, 7, 8, 9, 10, 11, 12, 13].

Referencing: Referencing follows the Indigenous Archives Collective Indigenous Referencing Guidance for Indigenous Knowledges in acknowledging knowledge creation to address and dismantle oppressive systems denying people creation of their own culture [14]. This work privileges Nation, Country, or Language group in the reference list, if that information is provided within the source being cited or is clearly provided [14]. This work does not assume a person's affiliation if it is not stated clearly [14].

## 2 Background

Research is an unsafe term for First Nations Peoples [5, 15]. In the context of the lands now known as Australia, scientific racism provided platform for invasion and ongoing colonisation [1, 13, 16, 17, 18, 19, 20, 21]. Scientific racism in colonial research established flawed belief in a biological and genetically inherent racial hierarchy [21, 22] which dehumanised First Nations Peoples. When dehumanised, ongoing invasion focused on extermination of First Nations Peoples, with exploitation as an embedded by‐product [23].

Scientific racism's flawed method and racist ideology [16] underpinned social, biological, and anthropological research in fields including Phrenology and Eugenics [16, 21, 24]. Devoid of consent, scientifically racist colonial research methods were imposed upon First Nations Peoples, being done on and to, rather than with and for [25, 26]. Overtly racist work oversaw immoral unethical research conducted upon bodies and bones [16, 17] of First Nations Peoples positioned as specimens, less than, and othered [16, 17, 27]. Endorsed by academics, First Nations oppression and trauma [16, 17, 28] was consolidated by published scientific racism across the land now known as Australia which ascribed primitive theology, poorly evolved family structures, and most primitive form of human to First Nations Peoples, whilst comparing to European man as a standard of perfection [17]. Scientific racism delivered erroneous results and indelible negative stereotypes through means of building Indigenous inferiority into methodology [17] to produce inferiorising conclusions of First Nations Peoples [17]. Colonial research facilitated colonisation of Australia with established power differentials [1, 5, 15, 18, 29, 30], justifying displacement from Country, genocidal interventions, and destruction of means of First Nations knowledge creation, dissemination, and preservation. Scientific racism in colonial research predicated exclusion of First Nations Peoples from political, economic, education, legal, and health system design, implementation, evaluation, and research [18]. ‘Eurocentric constructions of the racialised Other, the construction of whiteness and its subsequent embodiment, enabled the establishment of this far‐flung outpost of British imperialism’ [22].

As a tool of colonisation, scientific racism in colonial research set precedent for intellectual terra nullius [31], destroying Indigenous knowledge systems [18, 25] and normalising superiority of White people and culture [18, 32]. Truth‐telling about the history of research violence used in colonising the lands now known as Australia includes ‘salvaging data on a dying race for science’ [33], prioritising data collection over providing healthcare, which associates research and researchers with active participation in genocide. Research methods used today are a legacy of ongoing colonisation, skewed social viewpoints, and biased datasets from past scientifically racist studies [16]. Globally, mainstream research methods are driven by a Western scientific model that is informed by past scientific racism as well a Western value set for research; the ways research is valued, and the ways evidence is conceptualised. In a domestic context, research methods privileging Eurocentric value sets and means of knowledge development facilitate learnings and frameworks that drive and support Western knowledge systems, silencing First Nations ways of knowing, being, and doing [17, 18]. This perpetuates ongoing harms to First Nations Peoples. The United Nations Declaration of the Rights of Indigenous Peoples is contravened where First Nations Peoples lose control of maintaining knowledges systems [18], so as mainstream foot health researchers we need to understand and respect this. Mainstream foot health research needs to broaden intellectual investments in First Nations health, address power imbalances causing health inequities, and see First Nations Peoples as architects of health advancement [34].

Eurocentric healthcare systems founded and developed by mainstream research across the lands now known as Australia impose an 8–9 years less life expectancy for First Nations Peoples [35, 36], as well as a poorer quality of life, with the burden of disease experienced by First Nations Peoples 2.3 times the rate of non‐Indigenous Australians, and diabetes burden 6 times as high [37]. Systemic inequities in foot health research, aggregated epidemiological foot health statistics, teaching and learning [38], and service delivery contribute to this inequality [39, 40]. As foot health researchers, meaningfully working to support self‐determined First Nations ways of working is a means of redistributing power in First Nations‐led co‐design of foot health research, education, and health systems that centre culture in health and healing [41] and work for First Nations Peoples, as judged by First Nations Peoples. Research discourse shaped by white hegemony presents problems experienced by First Nations populations as a product of First Nations incapacity [42]. To revert from such victim‐blaming, non‐Indigenous capacity in anti‐racist research and service delivery needs development [43]; ongoing non‐Indigenous ignorance of, and inaction to, will only serve to maintain oppression whilst preventing unlearning of whose attitudes and ways of working need addressing to achieve First Nations equality [42]. Ways of working in foot health research to bring about epistemological, ontological, and axiological change need to privilege First Nations methodologies. Aboriginal Participatory Action Research (PAR) is an Indigenous Research Methodology informed by Indigenous Standpoint Theory developed by First Nations researchers, scientists, Elders, and Communities [44, 45, 46]. Aboriginal PAR aligns with First Nations empowerment and self‐determination, and with First Nations cultural recognition [46]. Methodology recognises First Nations sovereign knowledges and restores First Nations knowledge systems [46, 47]. In authentic co‐design of foot health research, power is redistributed to First Nations Peoples to lead work in which Indigenous methodologies are privileged and in which non‐Indigenous peoples are positioned as supportive learners. Research may then be relational to the physical and spiritual [48], constructing foot health knowledge aligned with First Nations value sets, ethics, and guidelines [46]. Authentic First Nations‐led co‐design needs to be applied within foot health research processes to create culturally responsive research projects that are safe for First Nations participants, and which prioritise needs and outcomes as determined by First Nations Communities. This empowers First Nations direction of design, implementation, and evaluation of foot health systems outputs and informs governance.

The primary aim of this work therefore is to follow Aboriginal PAR methodology [46] to document First Nations determined ways of working with and for First Nations Peoples in foot health research. This privileges First Nations ways of working in science and research which have been practised on the continent now known as Australia since time immemorial [49], to provide learning in decolonisation of the foot health research space. Aware that a majority of First Nations Peoples live in regional and urban areas, but also that rural areas are locations of poorer health outcomes for all Australians, including First Nations Peoples [50, 51], the secondary aim of this work is to utilise First Nations ways of working in research in investigating what foot health means to First Nations and non‐Indigenous health practitioners working in rural foot health, as well as their perspectives of the needs for and service barriers to providing good foot care with and for First Nations Peoples. Results of analysing data collected following ways of working described in this paper will be published in subsequent work. Accumulatively, this research with and for First Nations Peoples delivers transformative learning so that non‐Indigenous foot and lower limb health workforce and mainstream healthcare improve cultural responsiveness skills and ways of doing that work to eliminate racism from foot health, stop systems killing and traumatising First Nations Peoples, and improve upon the little non‐Indigenous providers know about authentic podiatry services with and for First Nations Peoples [40]. This research methods paper with accompanying commentary is part of work supporting First Nations‐led, culturally informed, Community‐driven solutions to foot disease. To this end, may future service delivery for First Nations individuals, families, and Communities be led in design, implementation, and evaluation by First Nations individuals, families, and Communities, and be supported by non‐Indigenous people.

## 3 Ways of Working

This research was led by Indigenous research methodology; Aboriginal PAR [46] underpinned by self‐determination and a First Nations Advisory Group. PAR is a transformative paradigm promoting social justice [52]. Aboriginal PAR integrates Indigenous epistemology, ontology, and axiology (the equivalent language for Indigenous ways of knowing, being, and doing) in Western knowledge systems and the dominant culture in the land now known as Australia [3, 46, 53, 54]. This study used Aboriginal PAR to privilege First Nations research priorities and to engage and privilege First Nations Peoples in research processes [55] and culturally valid and meaningful knowledge development [55]. Privileging Indigenous research methodology is to meaningfully contribute to reclamation, rebuilding, and reinstatement of self‐determined First Nations knowledge systems [18]. Aboriginal PAR leading this research seeks change from decades of historical and ongoing systemic racism perpetuating power imbalance and resultant health inequities and inequality [43]. In this work, value sets, philosophies, and ways of working are not combined equally. In the pursuit of equity in ways of doing research with and for First Nations Peoples and parity in health outcomes, First Nations leadership instils empowered, self‐determined control of ways of working. That is, privileging Indigenous Methodology and First Nations ways of working complimented by First Nations discretion in picking and choosing any parts of Western methods to be modified, if and when required, and repurposed for use. This work seeks appraisal as a true, correct, and beneficial result of a participatory process of First Nations empowered and led co‐design by First Nations Peoples.

## 4 Relationships

Not to be understated, this work was planned with and for First Nations families, Elders, and Community with whom there were, and still are, long‐established, mutually beneficial, and trusting relationships. These relationships existed well before plans of undertaking research work together evolved and were founded upon years of individual, family, and Community engagement, familiarity, and humility. Method privileged these relationships in leading the work, in decolonising research [20], and in rebutting ‘Historical White Institutions’ [56] ‘misappropriating Aboriginal Knowledge Systems by allowing non‐Indigenous people to lead and act as ‘Mission Managers’ over Aboriginal and Torres Strait Islander ways of knowing, being and doing’ [56]. Ways of working included non‐Indigenous researchers developing understanding of Aboriginal PAR and decentring whiteness as an epistemological norm and resultant hegemony [18, 57]. Positioning one's non‐Indigenous self as a learner, un‐learner, and re‐learner in relationships, with understanding that learning, un‐learning, and re‐learning is ongoing, underpinned non‐Indigenous support of First Nations‐led research ways of working.

## 5 Local First Nations Leaders

Local First Nations leaders invited non‐Indigenous researchers with whom there were existing, long‐standing, and trusting relationships onto Country and into Community to do work. This research responded to the need for equity in delivery, and equality in outcome, of foot care services as determined by First Nations individuals and families in the Darkinjung Community, and more broadly, diverse First Nations Peoples across Country. The study was positioned with First Nations governance established in the Darkinjung and Wiradjuri research ‘Footprints for Life’ [58], which this research extends upon. This study had First Nations leadership, being part of a PhD project with four First Nations people with expertise in First Nations health and research leading design, development, and implementation as research supervisors. These First Nations members of the research supervisory team formed a separate governance mechanism for this qualitative study, a self‐determined First Nations Advisory Group. The Advisory Group led ways of working true and responsive to Indigenous Research Methodology. Local First Nations leaders and First Nations Advisory Group leadership established what non‐Indigenous researchers needed to be thinking about, privileging, learning, unlearning, and relearning across all levels of work.

## 6 Overarching First Nations Governance

Ways of working in foot health research is guided by Australian Institute of Aboriginal and Torres Strait Islander Studies (AIATSIS) leadership in First Nations ethics and research mandated by the AIATSIS Act (1989) [59]. The AIATSIS Code of Ethics for Aboriginal and Torres Strait Islander Research [60] foregrounds principles of Indigenous self‐determination, Indigenous leadership, impact and value, and sustainability and accountability in ways of doing First Nations research [60]. The AIATSIS Code of Ethics for Aboriginal and Torres Strait Islander Research is informed by the United Nations Declaration on the Rights of Indigenous Peoples [61] and underpins National Health and Medical Research Council (NHMRC) ethical guidelines for research with First Nations Peoples. Two NHMRC guidelines; ‘Ethical conduct in research with Aboriginal and Torres Strait Islander peoples and communities: Guidelines for researchers and stakeholders 2018’ [62] and ‘Keeping research on track II 2018’ [63] were developed including The Lowitja Institute, AIATSIS, and NHMRC Indigenous Research Ethics Guidelines (IREG) Review Working Committee [64]. These two guidelines further inform ways of doing research with and for First Nations Peoples. They describe a central core value of Spirit and integrity surrounded by core values of what researchers need to learn and then foreground in all work: Cultural continuity, Equity, Reciprocity, Respect, and Responsibility [62, 63]. With research led by, and being with and for First Nations Peoples, ethics approval for work which centred place was sought from the Aboriginal Health and Medical Research Council of NSW (AH &MRC) Human Research Ethics Committee. Embedding NHMRC core values, AH & MRC holds research accountable to five key principles in meaningful, ethical, and culturally responsive work [65]. Privileging First Nations leadership in local ethics prioritises net benefits for Aboriginal Communities, Aboriginal Community control of research, cultural responsiveness, reimbursement of costs, and enhancing Aboriginal capacity [65]. Fundamentally, ways of working described by both local and overarching First Nations leadership included non‐Indigenous members of the research team working to redistribute power and to de‐centre the dominant Western academy; providing platform for First Nations voices to be listened to in the foot health research space.

## 7 Non‐Linear Lens

First Nations leadership embedded relational ways of working in this foot health research. From the outset, research was considered in terms of how it may be appraised by the Aboriginal and Torres Strait Islander quality appraisal tool [66] once the work was completed. This First Nations‐led tool was specifically developed to appraise health research with and for First Nations Peoples [66]. Positioning the tool as directive had non‐Indigenous researchers clearly thinking about authentic First Nations Community consultation and priorities, empowered First Nations leadership, First Nations cultural protocols relative to place, and Indigenous methodology in their ways of working. Consulting the appraisal tool before and then during work made it continually relational to ways of working. This guided ways of working that were not consistently linear progressive, but rather ways which paused, went back to the past, moved sideways for consideration and support, which considered transformative journeys of people involved, and which considered research practices and outcomes in the context of time passed since 1788. Continually re‐evaluating ways of working was not sequential nor straightforward but revolved around ongoing relearning which centred guidelines, appraisal tools, local First Nations leaders, and what Community are saying. This non‐linear lens consolidated a place of 2‐way learning and an undertaking of strengths‐based approaches which acknowledged and moved beyond practices that have that have contributed to genocide of First Nations Peoples [67].

## 8 Study Design

Ways of working privileged Aboriginal PAR [46] in cross‐sectional qualitative study design. Inclusive research development and consultation led by Darkinyung and Wiradjuri Elders, First Nations Advisory Group, and broader First Nations Community members regarding all levels and constructs of work chose semi‐structured interviewing, a frequently used technique of interviewing within qualitative healthcare research [68, 69], for this study. Semi‐structured interviewing following a constructivist phenomenological approach to generate data was well suited to the setting and research objectives [52, 70, 71, 72]. Such design facilitates rich open‐ended data inclusive of thoughts, feelings, beliefs, and lived experiences and can explore more personal and sensitive issues relating to health services [68]. In this research, First Nations leadership of semi‐structured interview guides developed talking points about what foot health means to First Nations and non‐Indigenous health practitioners, as well as their perspectives regarding needs for, challenges in accessing, and barriers to providing good foot health care with and for First Nations Peoples. Study design was planned and adapted as creating a more culturally responsive space that First Nations people are more likely to judge and experience as safe, and to translate study findings into sustainable changes in policy and practice that bring benefits to First Nations individuals, families, and Communities.

## 9 Study Participants and Selection Procedures

With ways of working grounded in Aboriginal PAR, First Nations Advisory group leadership and non‐Indigenous researchers developed strength‐based, culturally responsive research email communication, Participant Information Statement, Consent Form, and Withdrawal of Consent Form to create a research space that, from first interaction with the project, would be more likely to be deemed safe to approach and use by First Nations participants. Most importantly, this way of working clearly positioned First Nations leadership in research and decolonised the language used. This way of working minimised the risk of selection bias in this study where First Nations Peoples could hesitate to participate because of judged lack of safety in work and/or triggering of historical mistrust in research underpinned by scientific racism [1, 5, 13, 15, 16, 17, 18, 19, 20, 21, 73]. Potentially eligible participants were identified through professional clinical, Community, and research groups. Participants were sent initial emails inviting participation in the research. Semi‐structured interview sample size was *n* = 10. In mainstream research, qualitative sample sizes are commonly determined by reaching thematic saturation [68], deemed to have occurred when interviews fail to produce new information [74, 75]. Mainstream literature reports that highly meaningful healthcare research utilising semi‐structured interviewing may be completed with as few as 8–12 participants [68, 76, 77, 78], but does raise that in qualitative health research, sample size insufficiency is seen to threaten the validity and generalisability of studies' results; often in general scientific terms [79]. Mainstream method additionally advises involving sample size considerate of homogeneity of the sample [68, 78] and of epistemological and methodological influences [79, 80]. This work considers sample size in the context of Indigenous methodologies, where thematic saturation has no parallel, where there is an expectation that researcher/participant relationships found the work, and where these respectful, mutually beneficial, and foundational relationships are ongoing [81, 82]. Within the land now known as Australia, recent qualitative health research involving First Nations and non‐Indigenous researchers, First Nations participants, local First Nations‐led research sub‐committee endorsement for ethics approval, and semi‐structured interviewing rested on sample size being a result of the right mix of participants and enough data to convey a more complicated mosaic of multi‐faceted stories [83, 84, 85]. Ways of working guiding selection procedures in this work replicate this. Sample size is considerate of the diversity of First Nations Peoples. Ways of working do not homogenise working with and for over 240 First Language groups, cultures, lore, and ways of conducting science and research. Ways of working aim to deliver some overarching results, some points to be clearly considering, and some reproducible ways of working which may be used relative to place. Ways of working regarding selection procedures highlight ongoing relationships are of prioritised importance in working right ways in First Nations foot health research and that saturation of data judging finality and all there is to know on a topic, combined with homogenising generalisability to diverse populations, deems competence in another culture, positions it as less than, and reinforces epistemological power imbalance.

## 10 Participant Recruitment

Sampling that was purposive and seeking large variation was used to recruit health practitioners providing foot care with and for First Nations Peoples that was representative of the clinician population (based on the cultural aspects of identity and experience, and based on workplace setting). Health practitioners delivering services relating to foot care with and for First Nations Peoples were recruited to participate in semi‐structured interviews. Healthcare workforce eligible to participate in this aspect of the research needed to hold current registration with their discipline's professional registration board. The details of the study (Participant Information Statement, Consent Form, and Withdrawal of Consent Form) were issued well before interview dates, with informed written consent being obtained prior to conducting any interview. Ways of working directed that time be taken to allow participants to discuss participation with family, friends, Community, and workplace. Consent forms in this work were developed with First Nations Advisory Group leadership and contained the question ‘Are you Aboriginal and/or Torres Strait Islander?’ This question was prefaced with introduction to providing a subsequent culturally responsive interview space, to being able to report ethnicity in data, and to privileging First Nations voices and worldviews in the results of the work.

## 11 Data Collection

With First Nations Advisory Group leadership, the research team developed two series of open‐ended questions informed by Aboriginal PAR, First Nations Advisory Group expertise, and recent literature, which were used as guides during interviews. Responses to the question ‘Are you Aboriginal and/or Torres Strait Islander?’ determined which set of semi‐structured interview questions would be used during interviewing; questions for First Nations participants or questions for non‐Indigenous participants. Although similar, the difference in phrasing and positionality in sets of interview questions foregrounded Aboriginal PAR ways of working and cultural responsiveness in conducting interviews. Research interviews primarily followed a line of pre‐determined questioning, with allowance for follow‐up unrehearsed questions based upon participant responses during interviews. Because of this, and as part of culturally responsive ways of working in research, First Nations participants were also offered a First Nations member of the research team be present during data collection. This method aimed to empower First Nations Peoples' self‐determined participation in the work, aimed to address power imbalance in interviewing, and aimed to rebut the stigma of historical scientific racism.

Interview questions developed with First Nations Advisory Group leadership embedded avoiding critiquing care provision and, in the case of non‐Indigenous participation, avoiding speaking for First Nations Peoples. Interview questions developed with the First Nations Advisory Group were pilot tested with a First Nations research team member who was blinded to the interview guide before any data collection interviews were conducted. Data collection was conducted online with semi‐structured interviews all completed via Zoom Video Communications teleconference software at a time and place convenient to consenting participants. Participants consented to interviews being audio recorded using Zoom software and backed up with Dictaphone recordings, whilst additionally having field notes taken to inform later data analysis. All participants were given the opportunity to verify their data afterwards and were additionally given the opportunity to read final manuscript drafting prior to any submission for publication. This presented First Nations participants the opportunity to check that their data was accurate and had not been misconstrued or taken out of context during research process. It provided chance for First Nations participants to check that they were not misrepresented by the work and to build trust in culturally responsive ways of working in foot health research.

## 12 Data Sovereignty

This research supports the Maiam nayri Wingara Aboriginal and Torres Strait Islander Data Sovereignty Group, operating across the lands now known as Australia [86, 87]. Ways of working in this study negotiated rights to access First Nations existing cultural and intellectual property from the outset, with full disclosure and transparency including describing proposed ways of disseminating results. Founded in scientific racism, data on and about, not with and for First Nations Peoples has been weaponised against First Nations Peoples [87, 88], negatively stereotyping First Nations Peoples as a different, dysfunctional, and disadvantaged problem needing to be fixed [88, 89]. BADDR data are data which are Blaming, Aggregate, Decontextualised, and Deficit focused data of Restricted access [89]. BADDR data have resulted in oversupply of descriptive data of and about First Nations Peoples and under delivery of data evaluating efficacy of ways of working in First Nations health with and for First Nations Peoples [33, 90, 91, 92]. This systemically racist lever of ongoing colonisation has First Nations Peoples positioned as over‐researched and without corresponding improvements in health outcomes [33, 86, 87, 88, 89]. Paradigms need to shift from BADDR Data to Indigenous Data Sovereignty [86, 87, 88, 89]. Indigenous Data Sovereignty is ‘the right of Indigenous people to exercise ownership over Indigenous Data’ [86, 87], it is practiced through Indigenous Data Governance [86, 87]. Empowered, self‐determined Indigenous Data Governance is ‘the right of Indigenous peoples to autonomously decide what, how and why Indigenous Data are collected, accessed and used’ [86, 87]. As in this study, foot health research needs to align with Maiam nayri Wingara principles of Indigenous Data Sovereignty and Governance. Foot health research with and for First Nations Peoples must have data being controlled by and accessible to First Nations Peoples, and data must be relevant to, and respect First Nations self‐determined interests and governance [86, 87, 93]. Facilitating this, non‐Indigenous researchers need to yield space in research, develop cultural humility and anti‐racist skill sets, and support First Nations leadership to tell First Nations stories utilising First Nations ways of working in data management [86]. Foot health research with and for First Nations Peoples can then deliver outcomes meeting the needs, values, and aspirations of First Nations Peoples as evaluated by First Nations Peoples.

## 13 Data Analysis

First Nations‐led ways of working in data analysis included all transcripts being anonymised, (identifiable only by participant number and First Nations/non‐Indigenous voice) and transcribed verbatim by researchers manually. This better immersed researchers in data, improved their understanding and comprehension of phrasing and meanings of words used across many diverse First Nations, and removed impacts of software erroneously analysing data specific to First Nations languages, places, and foot care. For rigour and trustworthiness, ways of working included peer debriefing in code recording, 2‐way learning, being reflexive, and having themes cross‐checked by a First Nations researcher to identify areas of potential bias and to determine the reliability of the data interpretation [58, 94].

Transcriptions and field notes were read, categorised, and inductively coded by a non‐Indigenous researcher (JG) and were then reviewed by a First Nations researcher to minimise bias and cross‐cultural misinterpretation. Ways of working placed a non‐Indigenous researcher with existing relationships with First Nations leadership and a more developed culturally responsive and anti‐racist skillset (as judged by First Nations Peoples) in this role. The non‐Indigenous researcher (JG) additionally had experience in both mainstream and First Nations foot health services and research, as well as experience working on Wiradjuri and Darkinjung Country. Aboriginal PAR made it clearly understood that transcribing work is not a task that an inexperienced unfamiliar non‐Indigenous researcher can just walk into. It is also noted that multiple raters improve consistency between raw data and researcher interpretation in a phenomenological study [95, 96]. Preliminary analysis commenced with contextualisation of data by open coding [71, 72, 97], generating an initial broad coding framework. With agreement upon the framework from the research team, coding then progressed iteratively with new coded material being discussed at regular intervals and regrouped and/or condensed into categories as new data were gathered. Analytic induction utilised inductive reasoning and constant comparison method to identify common and overarching themes and perform thematic analysis [75, 98, 99, 100]. All authors agreed upon the final themes and thematic analysis included identifying similarities and differences within and between the interviews and extraction of relevant quotes. Demographic data was managed using Microsoft Excel. Having multiple researchers involved in teaching, learning and implementing ways of working, and being transparent in analytical processes, produced more robust and trusted research outcomes [71, 101, 102].

### 13.1 Ethics Approval

Ethical approval was granted by The Aboriginal Health and Medical Research Council (1376/18) and the University of Newcastle Human Research Ethics Committee (approval No. H‐2018 0035).

## 14 Discussion

The primary aim of this work was to document and discuss the development and implementation of First Nations‐led, culturally responsive ways of working in foot health research in a cross‐sectional qualitative study. These methods underpin research undertaken by the group to establish health practitioners' perspectives of foot health and needs for and challenges to providing good foot care with and for First Nations Peoples that will be published subsequently. This discussion further expands on the process of developing culturally responsive research methods privileging Aboriginal PAR and empowering First Nations leadership and ways of working in foot health research to create third spaces of combined knowledges [103]. As a guiding paper, this discussion names First Nations leadership of work across all levels as the key requirement in redistributing power to create an authentic First Nations‐led co‐designed research space. Empowered First Nations leadership may inform ways of working in foot health research which directs both teaching and practice, whilst rebutting Western hegemony and scientific racism and leading the way for change. First Nations‐led co‐design is a tool for social justice [104, 105], and in healthcare can reduce racism and improve access through its decolonising methods and strategically anti‐racist approaches [5, 10, 43]. There are specific requirements for such research process and there are very clear take home messages for foot health researchers from this work. Ways of being for non‐Indigenous foot health researchers need to come from a place of cultural humility [106, 107, 108]. As culturally humble, supportive non‐Indigenous researchers may better reposition power, prioritise First Nations‐led ways of knowing, being, and doing, and position one's non‐Indigenous self to begin learning, unlearning, and relearning. Time needs to be taken by non‐Indigenous researchers for work on self, where humility is fundamentally about recognising one's own non‐Indigenous systemic privilege and power, the harm and trauma it can cause, and the need to relinquish it so that it may be repositioned power prioritising First Nations‐led ways. Non‐Indigenous researchers need to take time to become comfortable sitting with discomfort, for being prepared to be self‐critical and uncomfortable through the learning, unlearning, and relearning, and to position any discomfort as negligible to that felt by First Nations Peoples impacted by scientific racism since 1788. Time also needs to be taken by non‐Indigenous researchers to build trusting, mutually beneficial, and two‐way learning relationships with Communities, who then lead and advise them during process [109].

As required for this work, time needs to be prioritised for learning from and with Communities on how to interact in culturally responsive ways. This needs to occur whilst being respectful of First Nations Peoples' time and competing priorities [7, 110] and without increasing colonial load [111] placed upon First Nations Peoples. Non‐Indigenous foot health researchers need to work to restore trust in process that aspires equity in foot health research contributing to equality in overall health outcomes across all First Nations. Such ways of working need to be considerate of the impacts of history [30, 112]. To assume that gradual inclusion of First Nations epistemology, ontology, and axiology [3, 46] on Western academic terms and under Western academic control will change systems which discriminate against First Nations Peoples is futile hope; whilst being complacent is being complicit. Citing the ongoing ‘racial violence within the Australian health system’ [113] and cycle of ‘Closing the Gap’ policy failures [10, 113, 114], it will take real non‐Indigenous accomplices and change makers [115] in places of foot health research to develop skills in ways of working which will re‐write design, implementation, and evaluation of foot health systems. Intellectual terra nullius [31] powered by scientific racism has removed First Nations science, research, and ways of knowing, being, and doing from foot health systems and colonising scientific racism in the foot health space is ongoing.

Internationally, the thrifty gene hypothesis, formally rejected in 2011, is an example of ongoing failed scientific racism which proposed Indigenous Peoples were genetically predisposed to Type 2 diabetes [116, 117]. With implications of manifesting flawed conceptualisation locally, such victim‐blaming scientific racism ignores impacts of colonisation being bad for your health [30], ignores racism impacting overall health and wellbeing [9, 46, 118], and ignores displacement from Country and its successful ongoing health paradigm as causes of poorer Indigenous health outcomes; including diabetes‐related foot disease. Such erroneous work, and its subsequent dissemination by Western academia, contributes to deflection of root cause of health inequities away from systemic racism, and to ongoing exploitation and harm in ways of doing foot health research. Decolonisation [46] relational to the way we value research, methods and outcomes, and in addition, to the ways we work in foot health is required. First Nations‐led research takes back self‐determined ways of working, empowers, heals, and centres culture in action and process, as well as in outcomes of work [18, 46]. Western hierarchy of evidence ranks cross‐sectional qualitative study design relatively low [119]. However, this work raises contention that the voices, results, and discussion here and those amplified, presented, and discussed using Indigenous methodologies in foot health research led by First Nations experts, may be more impactful and meaningful than mainstream randomised controlled trial reporting. This work questions the following: Is non‐Indigenous, mainstream value set for research working? Therefore, First Nations‐led co‐design of foot health research becomes foundational to informing non‐Indigenous researchers in particular, across many mainstream study designs, how to do it properly, how to work right ways, and how to work different ways, to achieve different outcomes; good foot health services providing good foot health outcomes with and for First Nations Peoples. The simple explainer here is that this is hard work. Hard work is required from non‐Indigenous researchers in undertaking ongoing cultural responsiveness learning, unlearning, and relearning, and part of this education is understanding that it is hard work for First Nations Peoples to teach and guide without process contributing to excessive colonial load. Colonial load unfairly burdens First Nations Peoples with having to justify First Nations ways in colonised processes [111] and is compounded by time spent, emotional energy expended, triggering content, and non‐Indigenous ignorance [43]. This discussion therefore is explicit in positioning non‐Indigenous researchers as the people needing to take on the hard work. This study informs readers to better understand process and the relationships behind it, as well as nuances. Readers may rethink accepted process, and how they interpret, use, and place value on results. Without centring First Nations voices and worldviews, without self‐reflection of positioning to colonial power, and without understanding the biased conditions of ongoing colonisation [120], foot health research and its methods will maintain Western hegemony, inequities in health systems, and unequal health outcomes for First Nations Peoples. Non‐Indigenous foot health researchers can be part of the solution or can be part of the problem.

### 14.1 Strengths and Limitations

Strengths are that this research benefited the participants and First Nations Communities by privileging their voices and ways of working in research with and for. The research demonstrates capacity strengthening for both First Nations and non‐Indigenous individuals from involvement in the work, developing implementation of Aboriginal PAR, and empowering authentic co‐design in the research space. Non‐Indigenous researchers improving skills in taking time, deep listening, supporting First Nations leadership, challenging the norms of systemic research inequities, and developing decolonising ways of doing research which redistribute power are further strengths of this work. Importantly, everyone involved in the research had opportunities to learn from each other, to experience First Nations ways of working, to display cultural humility [106, 107] and self‐reflexivity [121], and to develop skills towards creating a culturally responsive research space [46, 55] where First Nations Peoples are welcomed, believed, supported, and do not experience racism in any form [3, 122, 123]. A strength of this research was its negotiated rights to access First Nations existing cultural and intellectual property, but a major limitation exists in the need for improved measures and mechanisms protecting First Nations ownership of data research created and seeing to First Nations Peoples having control over the collection and management of research materials [66]. Ways of Western university dominance of a PhD project, of which this work is a part, need to be addressed to demonstrate true power redistribution facilitating First Nations self‐determined decision making regarding caring for, accessing, storing, protecting, and controlling the use of data for the entirety of its lifecycle [124].

## 15 Conclusion

This work documents First Nations‐led ways of working in foot health research and brings First Nations‐led ways of working in cross‐sectional qualitative study design to published literature. This study provides qualified voiced lived experience which foot health research must listen to and receive direction and learning from. Since invasion, foot health research has been socialised into dominant colonial research paradigms conjuring positionality of First Nations Peoples as a problem, rather than working with and for First Nations Peoples. Authentic First Nations‐led co‐design of research delivers ways of working to change systems of oppression. Anti‐racist and cultural capability skill sets of those working in mainstream foot health research can develop to build culturally responsive spaces for First Nations Peoples to approach, use, and lead in, and which inform of culturally safer solutions to foot disease as judged by First Nations Peoples. Culturally safer services need to be founded on decolonised research evidence led by First Nations Peoples. Aboriginal and Torres Strait Islander Peoples' access to healthcare is reduced by lack of culturally safe service [38, 49, 125]. In Communities where provision of healthcare, including foot care is culturally safe, Aboriginal and Torres Strait Islander Peoples access services more frequently and have better health outcomes [38, 40, 126].

## Author Contributions

**James Gerrard:** project administration, data curation, formal analysis, writing–original draft, writing–review and editing. **Shirley Godwin (Badimaya Yamatji):** supervision, validation, data curation, formal analysis, visualization, methodology, writing–review and editing. **Kim Whiteley (Wiradjuri):** supervision, validation, formal analysis, visualization, methodology. **James Charles (Kaurna):** supervision, validation, formal analysis, visualization, methodology. **Sean Sadler:** writing–review and editing. **Vivienne Chuter:** supervision, data curation, formal analysis, visualization, methodology, writing–review and editing.

## Funding

The authors have nothing to report.

## Ethics Statement

Ethical approval was granted by The Aboriginal Health and Medical Research Council (1376/18) and the University of Newcastle's Human Research Ethics Committee (approval No. H‐2018 0035). All participants provided written informed consent.

## Consent

The culturally responsive Participant Information Statement received by all participants included information that de‐identified findings would be used in a publication.

## Conflicts of Interest

The authors declare no conflicts of interest.

## Data Availability

The datasets generated and/or analysed during the current study are not publicly available respecting Indigenous Data Sovereignty. They are available from the corresponding author on reasonable request.
